# Corticofugal and Brainstem Functions Associated With Medial Olivocochlear Cholinergic Transmission

**DOI:** 10.3389/fnins.2022.866161

**Published:** 2022-04-27

**Authors:** Felipe Munoz, Sergio Vicencio-Jimenez, Pascal Jorratt, Paul H. Delano, Gonzalo Terreros

**Affiliations:** ^1^Instituto de Ciencias de la Salud, Universidad de O’Higgins, Rancagua, Chile; ^2^Universidad de Valparaíso, Valparaíso, Chile; ^3^Department of Otolaryngology-Head and Neck Surgery, The Center for Hearing and Balance, Johns Hopkins University School of Medicine, Baltimore, MD, United States; ^4^National Institute of Mental Health, Klecany, Czechia; ^5^Third Faculty of Medicine, Charles University, Prague, Czechia; ^6^Facultad de Medicina, Neuroscience Department, Universidad de Chile, Santiago, Chile; ^7^Department of Otolaryngology, Hospital Clínico de la Universidad de Chile, Santiago, Chile; ^8^Centro Avanzado de Ingeniería Eléctrica y Electrónica, AC3E, Universidad Técnica Federico Santa María, Valparaíso, Chile; ^9^Facultad de Medicina, Biomedical Neuroscience Institute, Universidad de Chile, Santiago, Chile

**Keywords:** auditory efferent, cholinergic, α9-knock-out mice, auditory, olivocochlear

## Abstract

Cholinergic transmission is essential for survival and reproduction, as it is involved in several physiological responses. In the auditory system, both ascending and descending auditory pathways are modulated by cholinergic transmission, affecting the perception of sounds. The auditory efferent system is a neuronal network comprised of several feedback loops, including corticofugal and brainstem pathways to the cochlear receptor. The auditory efferent system’s -final and mandatory synapses that connect the brain with the cochlear receptor- involve medial olivocochlear neurons and outer hair cells. A unique cholinergic transmission mediates these synapses through α9/α10 nicotinic receptors. To study this receptor, it was generated a strain of mice carrying a null mutation of the Chrna9 gene (α9-KO mice), lacking cholinergic transmission between medial olivocochlear neurons and outer hair cells, providing a unique opportunity to study the role of medial olivocochlear cholinergic transmission in auditory and cognitive functions. In this article, we review behavioral and physiological studies carried out to research auditory efferent function in the context of audition, cognition, and hearing impairments. Auditory studies have shown that hearing thresholds in the α9-KO mice are normal, while more complex auditory functions, such as frequency selectivity and sound localization, are altered. The corticofugal pathways have been studied in α9-KO mice using behavioral tasks, evidencing a reduced capacity to suppress auditory distractors during visual selective attention. Finally, we discuss the evolutionary role of the auditory efferent system detecting vocalizations in noise and its role in auditory disorders, such as the prevention of age-related hearing loss.

## Introduction

Acetylcholine is an important neurotransmitter for both the maintenance of internal homeostasis and the interaction of individuals with the external environment (Picciotto et al., 2012). Several physiological functions depend on cholinergic transmission, including immunological, endocrine, and neural responses (Picciotto et al., 2012; Cox et al., 2020). In the nervous system, cholinergic transmission is ubiquitous, including, for example, peripheral synapses that regulate autonomic and motor responses, and central connections that modulate sensory and cognitive functions (Huang et al., 2000; Miles et al., 2007; Zagoraiou et al., 2009; Jordan et al., 2014; Sourioux et al., 2018; Parikh and Bangasser, 2020). Due to the immense diversity of neural circuits that depend on cholinergic transmission, the specificity of cholinergic receptors at the synaptic level is essential for the selectivity of their functions.

Cholinergic transmission is mediated *via* muscarinic and nicotinic receptors, which involve metabotropic and ionotropic signaling, respectively (Ishii and Kurachi, 2006; Hurst et al., 2013). Regarding the auditory system, there are efferent pathways connecting the brain with the cochlear receptors, and in the final synapses of these descending circuits, the auditory efferent system (AES) possesses a unique type of cholinergic transmission that has evolved in vertebrates. These connections are mediated by α9/α10 nicotinic acetylcholine receptors (nAChRs), located in the synapses between medial olivocochlear neurons (MOC) and outer hair cells (OHC) of the cochlea (Elgoyhen et al., 1994, 2009; Delano and Elgoyhen, 2016).

In Vetter et al. (1999) generated a strain of mice carrying a null mutation of the Chrna9 gene, giving rise to α9-KO mice, which lack cholinergic transmission between MOC and OHCs. These genetically modified mice provided a unique opportunity to study the role of MOC cholinergic transmission in auditory and cognitive functions.

This article reviews behavioral and physiological studies examining the role of cholinergic MOC synapses in auditory and cognitive functions, emphasizing those performed in α9-KO mice. We also discuss the possible evolutionary role of the auditory efferent system in mammals, probably as a feedback loop to enhance the detection of acoustic signals in noise. Finally, we present evidence that involves the MOC cholinergic transmission in auditory disorders, such as age-related hearing loss.

## Auditory Efferent System

**FIGURE 1:**
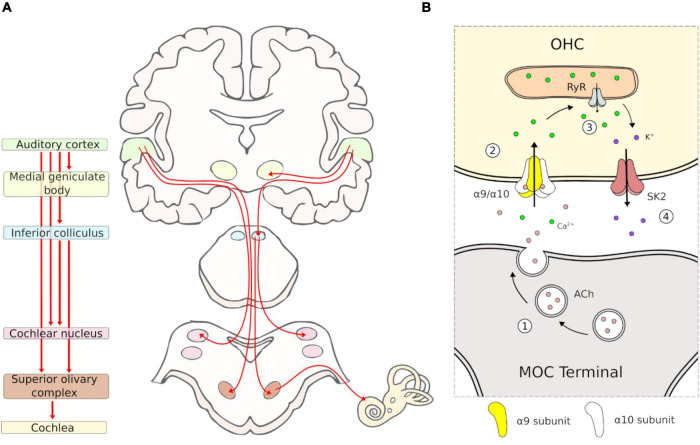
Schematic representation of auditory efferent system pathway and MOC-OHC synapse. **(A)** Diagram of the auditory efferent system. Efferent pathways are depicted in red arrows. The auditory cortex, medial geniculate body, inferior colliculus, cochlear nucleus, superior olivary complex and cochlea are depicted with the same color in the left and right panel. **(B)** Diagram of the MOC-OHC synapse. Acetylcholine (ACh) is released from the MOC terminal (1) and binds the postsynaptic α9/α10 receptor, which produces a Ca^2+^ influx (2). Then, the calcium-induced calcium release by ryanodine receptor (RyR) in the synaptic cistern (3) activates SK2 channels with the subsequent K^+^ efflux (4), producing a hyperpolarization of the OHC.

The auditory efferent system is a neural network that originates in the auditory cortex and projects to multiple subcortical nuclei of the central auditory pathways. These corticofugal pathways generate several feedback loops, including: the (i) collicular-thalamic-cortico-collicular- loop; (ii) cortico-(collicular)-MOC circuit; and (iii) cortico-(collicular)-cochlear nucleus loop (Terreros and Delano, 2015). The most peripheral section of the AES pathways projects from the superior olivary complex in the brainstem to the inner ear and auditory nerve, via MOC and lateral olivocochlear neurons, respectively (AES pathways are summarized in Figure 1A).

The MOC system appears to be present in all mammals (Smith et al., 2005). Comparative studies suggest that inner ear efferents emerged during evolution from facial branchial motor neurons, which project to the inner ear instead of facial muscles (Fritzsch and Elliott, 2017). Like motor neurons, MOC neurons release acetylcholine as their main neurotransmitter activating nicotinic receptors in the OHCs. Pharmacological studies on MOC-OHC synapses have shown that auditory efferent effects at the cochlear receptor are mainly mediated by the α9/α10 nicotinic cholinergic receptors (nAChRs) located in the basolateral domain of OHCs (Kujawa et al., 1992, 1994; Rothlin et al., 1999; Vetter et al., 1999; Elgoyhen et al., 2001). The activation of α9/α10 nAChRs by acetylcholine produces an increase of intracellular Ca^2+^ concentration, permitting the opening of K^+^ channels (SK2) at the basolateral domain, followed by an outward current that hyperpolarizes the OHCs (Figure 1B). The physiological effect of this OHC hyperpolarization is the reduction of basilar membrane motion and an overall cochlear sensitivity decrease (Elgoyhen and Katz, 2012). It is important to emphasize that, given its position at the final synapses of the auditory efferent network, studying the role of the α9/α10 nAChRs is paramount to understanding the AES function.

## The α9/α10 Nicotinic Acetylcholine Receptors

The evolutionary history of the nAChRs can be traced back as far as a billion years (Fritzsch and Elliott, 2017), being ancestral even to multicellular animals. During the early evolution of animals, these receptors underwent rapid diversification into several subunits (Li et al., 2016). Specifically, Chrna9 subunits appear to be exclusively associated with vertebrates and its research history formally begins in 1994 (Elgoyhen et al., 1994). This receptor was identified showing a preferential localization in the cochlear hair cells of the vertebrate inner ear (Elgoyhen et al., 1994). In addition, it has also been found in dorsal root ganglia and in other non-neural tissues, i.e., mice lymphocytes and keratinocyte, rat alveolar macrophages, and murine bone marrow cells (Lips et al., 2002; Peng et al., 2004; Chernyavsky et al., 2007; Colomer et al., 2010; Lee et al., 2010; Mikulski et al., 2010; Chikova and Grando, 2011; Koval et al., 2011; Hollenhorst et al., 2012; Zablotni et al., 2015; Jiang et al., 2016; St-Pierre et al., 2016), illustrating possible physiological functions in nociception and beyond the nervous system.

Although the cholinergic nature of MOC was known since the late 1950s (Churchill and Schuknecht, 1959), the structure of this cholinergic receptor remained unknown for almost four decades. This receptor is a pentameric cation channel composed of two α9 and three α10 subunits with a nicotinic-muscarinic pharmacological profile (Elgoyhen et al., 1994, 2001; Plazas et al., 2005). The α10 subunit of the OHC nicotinic receptor was cloned in Elgoyhen et al. (2001), while Vetter et al. (2007) demonstrated that both subunits (α9 and α10) are required for a functional channel. These authors concluded that the presence of the α10 subunit of nAChR is essential for MOC functioning (Elgoyhen et al., 1994, 2001; Vetter et al., 1999; Weisstaub et al., 2002).

## α9-KO Mice

In Vetter et al. (1999) generated a strain of mice carrying a null mutation of the Chrna9 gene, giving rise to α9-KO mice. This mouse was developed by replacing exon 4, which contains the coding sequence of the ligand-binding site and its surrounding sequences of the intron of the Chrna9 gene, with a neomycin resistance gene. This translates into a nonfunctional α9 subunit, allowing investigations of the α9- nAChR *in vivo*.

Despite no evident abnormalities in the gross cochlear morphology of α9-KO mice, as compared to wild type (WT), including the cochlear duct, hair cells, supporting cells, and spiral ganglion neurons (Vetter et al., 1999), several abnormalities have been described in the morphology and number of synaptic terminals between MOC neurons and OHCs. Specifically, larger and fewer MOC synaptic terminals have been described in α9-KO mice (Vetter et al., 1999). For instance, in the middle cochlear turn of WT mice, most of the OHCs are contacted by two efferent terminals while in the α9-KO mice, most OHCs are contacted by a single efferent terminal. This evidence indicates that synaptic development of MOC neurons is altered in the α9-KO mice, raising a caveat for the interpretation of these results.

## Auditory Function in the α9-KO Mice

As evaluated by behavioral detection of tones in quiet and background noise conditions, hearing thresholds are normal in the α9-KO mice (Prosen et al., 2000; May et al., 2002). Similarly, electrophysiological assessments using wave V thresholds of auditory brainstem responses (ABR) have confirmed the presence of normal hearing thresholds in the α9-KO mice compared to WT mice (Terreros et al., 2016). As expected, MOC function is abolished in the α9-KO mice when evaluated by electrical stimulation of MOC fibers at the floor of the fourth ventricle (Vetter et al., 1999), and diminished when assessed with contralateral sound stimulation and measuring auditory-nerve responses through wave I from ABR (Terreros et al., 2016).

Other auditory alterations have been found using the prepulse inhibition of the Acoustic startle response, as it is decreased in the α9-KO mice and increased in mutant mice that have an enhanced MOC function (L9’T-KI) (Taranda et al., 2009; Allen and Luebke, 2017; Lauer et al., 2021). Furthermore, the α9-KO mice exhibit deficits in sound localization tasks, as evaluated in conditioned lick suppression tasks to assess the minimum audible angle (Clause et al., 2017). Evidence shows that frequency selectivity is also impaired in mice models lacking MOC transmission, as suggested by electrophysiological and behavioral studies (Clause et al., 2014, 2017). In sum, the lack of MOC cholinergic transmission does not alter hearing thresholds, affecting, however, more complex auditory functions, such as pre-pulse inhibition, frequency selectivity and sound localization. Changes in auditory function in α9-KO are summarized in Table 1.

**TABLE 1:** Overview of auditory studies in α9-KO.

Auditory function	α 9-KO	References
MOC synaptic terminals per OHCs	Lower number of efferent contacts and greater volumen	Vetter et al., 1999
Tone and intensity discrimination	Normal in quiet and background noise	Prosen et al., 2000; May et al., 2002
Prepulse inhibition threshold	Decreased in quiet, but normal in background noise	Allen and Luebke, 2017
Sound localization in conditioned lick suppression task	Deficits in minimum audible angles	Clause et al., 2017
Contralateral noise suppression of ABR waves I	Decreased magnitude	Terreros et al., 2016
**Corticofugal pathway**		
Two-choice visual discrimination task with auditory distractors	Fewer correct responses and more omissions during the presentation of 65 dB clicks and tones	Terreros et al., 2016
	Strength of the olivocochlear reflex correlates with the correct responses and omissions	Terreros et al., 2016
**Brainstem olivocochlear**		
**Protection to acoustic trauma**		
ABR threshold after noise exposure	Permanent elevation	Boero et al., 2018
IHC ribbon synapses after noise exposure	Decrease in number of synaptic puncta in basal cochlear area	Boero et al., 2018

*Auditory functions of α9-KO compared with wild type mice.*

## Auditory Efferent Corticofugal Pathways

One of the proposed functions of the AES is the suppression of irrelevant auditory distractors during visual attention. This hypothesis emerges from studies performed in behaving cats and chinchillas during visual selective attention tasks, in which the animals showed a reduction of auditory nerve responses to distracting sounds (Oatman et al., 1971; Delano et al., 2007). This idea was tested in α9-KO mice that were trained in a two-choice visual discrimination task with auditory distractors (Terreros et al., 2016). In this task -similar to that used previously in chinchillas-, α9- KO mice made fewer correct responses and more omissions than WT mice when using 65 dB clicks and tones as distractors. On the other hand, when presenting broad-band noise at 90 dB as distractors, there were no differences between α9-KO and WT mice. Furthermore, the strength of the MOC reflex was positively correlated with the number of correct responses and negatively correlated with omitted trials in mice and chinchillas (Terreros et al., 2016; Bowen et al., 2020). As a conclusion, we propose that MOC activation aids in ignoring distracting sounds at moderate sound pressure levels, while middle ear muscle activation might help in suppressing auditory distractors at high sound pressure levels.

Recent works in humans and chinchillas have raised the hypothesis that visual working memory could also recruit MOC neurons. In this line, Marcenaro et al. (2021) indicated that the strength of MOC activation by contralateral sounds is enhanced during a visual working memory task in humans. In a recent work, Vicencio-Jimenez et al. (2021) studied late responses, executed 2.5 seconds after stimulus offset, in a visual discrimination task in chinchillas, in which they had to hold the visual stimulus in the working memory buffer to respond correctly. Late responses were correlated with the strength of the MOC reflex (contralateral sound) only when studied with auditory distractors, but not when visual discrimination was performed in silence (Vicencio-Jimenez et al., 2021). Together, these studies suggest that the activation of MOC neurons is a common characteristic of visual attention and visual working memory to suppress irrelevant sound during these cognitive tasks.

## Brainstem Olivocochlear Function and Auditory Pathologies

The MOC reflex involves brainstem circuits, and its activation reduces the cochlear gain, in a physiological effect that can be useful protecting against acoustic trauma and aging. In this line, the strength of the MOC reflex has been correlated with the susceptibility to noise-induced hearing loss (NIHL) (Maison and Liberman, 2000). This finding suggested that strengthening the MOC feedback could prevent hearing loss after noise exposure. Taranda et al. (2009) used the L9’T-KI mice with enhanced MOC function to confirm the idea that brainstem MOC feedback can reduce the damage induced by acoustic trauma.

Age-related hearing loss or presbycusis is a highly prevalent condition in elderly people, especially in individuals chronically exposed to acoustic noise. The disorder is characterized by reduced hearing sensitivity and speech understanding in noisy environments, altered central auditory processing, and a higher risk for developing cognitive impairment and dementia (Panza et al., 2015). On this basis, the strength of the efferent reflex has been linked to the prevention of the development of hearing loss, cochlear synaptopathy and age-related hair cell loss (Boero et al., 2018, 2020).

Therefore, enhancing MOC feedback arises as a promising approach to prevent age-related hearing loss. In this context, the α9/α10 nAChR offers varied opportunities to be a therapeutic target in the future. Two molecules known for being able to enhance the activity of this receptor are ascorbic acid and ryanodine (Zorrilla De San Martín et al., 2007; Boffi et al., 2013), opening the possibility of investigating their effects to prevent presbycusis. Although clinical evidence is limited, there is at least one report in humans showing a correlation between ascorbic acid intake and improved hearing in the older population (Kang et al., 2014). High-quality clinical trials are necessary to further investigate these molecules as treatments for age-related hearing loss.

The prevention or treatment of NIHL could also be intervened by pharmacological modulation of α9/α10 nAChR. Like by presbycusis, drugs that augment the effect of the MOC system on the OHCs could be used to prevent NIHL in workers performing in noisy conditions. Exposure to loud noise has short and long-term consequences since there may be a transient attenuation of hearing acuity or a permanent threshold shift (Le et al., 2017). However, there are occasions when exposure to loud noises generates an increase in hearing thresholds in frequencies that are not measured through conventional audiometry (Conventional audiometry measures up to 8 kHz, therefore frequencies between 8 and 20 kHz are not routinely studied). It has been proposed that the increase of the hearing thresholds in frequencies above 8 kHz could reflect hidden hearing loss (HHL) in humans, known as cochlear synaptopathy in animal models (Kujawa and Liberman, 2009).

## Evolutionary Role of Auditory Efferents

Despite the evidence supporting an important role for MOC cholinergic transmission in protecting against acoustic trauma and cochlear synaptopathy, it is unlikely that this was a critical factor in the evolutionary history of the AES. It is far more probable that its evolution is linked to its function with hearing in noise. The reason is that high-intensity noise that induces acoustic trauma is not common in natural conditions, making it more likely that this function arose as an exaptation or evolutionary spandrel (Gould, 1997; Smith and Keil, 2015). If we consider this evolutionary context, some interesting questions about this receptor arise. How has it changed in different mammals? What impact has the evolutionary history of different mammalian families had on the OHC nAChRs? For example, given the role of the MOC system in the regulation of cochlear gain, it is likely that it plays a part in the suppression of the individual’s own vocalizations, protecting the cochlea from overstimulation (Lauer et al., 2021). This would make it plausible to observe adaptations in the α9/α10 nAChR associated with animals that have high-intensity types of vocalizations, such as bats, cetaceans, and some primates.

In this context, future research in the α9-KO mice could evaluate the differences in vocalization patterns between them and WT mice. Furthermore, in the case of animals with high-intensity vocalizations, such as bats that vocalize above 100 dB (Moss and Schnitzler, 1995), protection against acoustic trauma might be a function directly selected in the MOC system. Therefore, it also seems feasible to find adaptations in the receptor associated with a high sound intensity environment.

## Conclusion

In conclusion, experimental models such as the Chrna9 KO mouse have allowed the development of multiple lines of auditory research, facilitating substantial advances in the knowledge about AES functioning, providing therapeutic possibilities for the treatment of auditory pathologies. Notwithstanding all the advances that the Chrna9 KO mouse has permitted in the study of auditory physiology, we believe that the development of a time-dependent conditional knock-out is key to the future understanding of AES role in audition and cognition. This type of tool would allow a better control of the possible compensatory effects on embryonic development or neurotransmitter plasticity due to the lack of cholinergic transmission (e.g., GABA), and to rule out the impact of non-neural tissues that are also affected in α9-KO mice.

## Author Contributions

FM and GT: original idea. FM, GT, SV-J, PJ, and PHD: manuscript writing. PJ: figure editing. FM, GT, and PHD: manuscript editing. All authors contributed to the article and approved the submitted version.

## Conflict of Interest

The authors declare that the research was conducted in the absence of any commercial or financial relationships that could be construed as a potential conflict of interest.

## Publisher’s Note

All claims expressed in this article are solely those of the authors and do not necessarily represent those of their affiliated organizations, or those of the publisher, the editors and the reviewers. Any product that may be evaluated in this article, or claim that may be made by its manufacturer, is not guaranteed or endorsed by the publisher.
